# Retraction of “Fabrication of Oro-Dispersible Sodium Valproate-Loaded Nanofibrous Patches for Immediate Epileptic Innervation”

**DOI:** 10.1021/acsbiomaterials.5c00432

**Published:** 2025-04-03

**Authors:** Ece Guler, Humeyra B. Yekeler, Zarife N. Ozdemir Kumral, Gita Parviz, Gul S. Ozcan, Burcu Uner, Sinem G. Demirbas, Simge Ayyildiz, Yusufhan Yazir, Deepak Kalaskar, Muhammet E. Cam

The authors retract the article due to duplication of content (Figure 4a) published in Figure 2a of https://doi.org/10.1016/j.ijbiomac.2023.128635 to avoid any misleading conclusions and to maintain scientific integrity.

The original article was published on December 21, 2024, and retracted on April 3, 2025.

